# Liquid Marble as Bioreactor for Engineering Three-Dimensional Toroid Tissues

**DOI:** 10.1038/s41598-017-12636-5

**Published:** 2017-09-28

**Authors:** Raja K. Vadivelu, Harshad Kamble, Ahmed Munaz, Nam-Trung Nguyen

**Affiliations:** 1 School of Natural Sciences, Nathan Campus, Griffith University, 170 Kessels Road, Nathan, QLD 4111 Australia; 2 Queensland Micro- and Nanotechnology Centre, Nathan Campus, Griffith University, 170 Kessels Road, Nathan, QLD 4111 Australia

## Abstract

Liquid marble is a liquid droplet coated with hydrophobic powder that can be used as a bioreactor. This paper reports the three-dimensional self-assembly and culture of a cell toroid in a slow-releasing, non-adhesive and evaporation-reducing bioreactor platform based on a liquid marble. The bioreactor is constructed by embedding a hydrogel sphere containing growth factor into a liquid marble filled with a suspension of dissociated cells. The hydrogel maintains the water content and concurrently acts as a slow-release carrier. The concentration gradient of growth factor induces cell migration and assembly into toroidal aggregates. An optimum cell concentration resulted in the toroidal (doughnut-like) tissue after 12 hours. The harvested cell toroids showed rapid closure of the inner opening when treated with the growth factor. We also present a geometric growth model to describe the shape of the toroidal tissue over time. In analogy to the classical two-dimensional scratch assay, we propose that the cell toroids reported here open up new possibilities to screen drugs affecting cell migration in three dimensions.

## Introduction

Culturing cells in a three-dimensional (3D) format has been attracting attention from the research community due to the wide range of applications such as drug screening^1^, high-throughput chemical analysis^2^, disease models^3^ and, particularly cell transplantation for injury repair^4^. There is an urgent need for a technology that enables cells to grow in three dimensions in their native state without the restriction of supporting scaffolds, thus closely mimicking the natural *in-vivo* environment^5^. Currently, the most popular scaffold-free microfluidic concept for a 3D cell culture is growing spheroids in hanging drops^6^. Recently, high-throughput screening with cell spheroids has been achieved using the hanging drop concept^7^ and non-adhesive microwell arrays^8^. However, challenges remain for growing tissues with complex shapes^9^ such as toroids^10–12^. Whilst each of previously reported scaffold-free methods is relatively easy to implement, they all have performance limiting factors. For example, hanging as well as sessile droplets are exposed to the atmosphere and evaporate quickly^13,14^. Due to the evaporation, the culture medium disappears within hours and sets a time limit on the culturing process. This bottle neck will be solved, if the culture environment could be maintained for a much longer period.

Liquid marbles, liquid droplets coated with hydrophobic powder, have been recently used for culturing cells^15^. Evaporation of the culture medium still is a major problem of liquid marbles as a bioreactors. Sessile liquid marbles on a solid surface evaporate and collapse within hours^14^ and are not suitable for culturing cells over days and weeks. We have solved this problem previously by floating the marble on another liquid^16–18^. The proximity to the liquid surface allows floating liquid marbles to maintain their integrity for days and weeks. This unique property makes floating liquid marbles extremely attractive for serving as a digital microfluidic bioreactor platform. Culturing cell spheroids has been successfully demonstrated in this system^19^. Moreover, a liquid marble can mimic the 3D microenvironment for cell growth. Adding drugs or soluble factor to the liquid marble can particularly influence self-assembly of cells to form larger aggregates.

The present paper reports another unique method to make a slow-evaporating liquid marble suitable for culturing 3D cell toroids. To date, the most common methods to engineer cell toroids are moulding with micro fabricated platform^10^, micro moulded hydrogels^11^ and non-adhesive conical pegs^12^. The mould allows the cells to aggregate into the toroidal shape. In this paper, we present a new method to allow cells to assemble by chemotaxis in a concentration gradient of growth factor. The key novelty of our method is the inclusion of a hydrogel sphere in the liquid marble. The hydrogel sphere serves as a storage of growth factor for slow release into the culture medium for sustainable growth of the 3D tissues. This platform offers additional controllability through careful manipulation of the marble motion, shape and composition of the hydrogel sphere, which in turn generates a concentration gradient of growth factor for chemotaxis. This platform allows for the growth of not only conventional cell spheroids but also more complex tissue geometries such as cell toroids. Cell toroids are tissues with a “doughnut-like” toroidal shape.

To date, drug screening for studying cell migration is predominantly carried out in a two-dimensional (2D) environment^20^. Cell migration induced by drug or growth factor has been examined by simple 2D scratch migration assays or single-cell assays, which may not accurately replicate the 3D *in-vivo* environment^21,22^. In contrast, a 3D tissue model has recently gained increasing interest in studying cell migration^23,24^, regeneration^25^, and repair^26^. A 3D model matches the mammalian tissue niche morphologically and physiologically. At the cellular level, a 3D environment supports complex cell-matrix interaction and maximizes the cell-cell interaction^27^ that closely emulate what cells in native tissue enviorment experience^28^. Additionally, the reorganization of actin cytoskeletal and cell–matrix adhesion is prerequisite for cells to adhere to the matrix and to exert contractile force to move forward. The cytoskeletal mediated tension in a 3D model leads to different migration speeds and patterns than that of a 2D model^28^. Moreover, scaffold-free 3D cultures are comprised of cells in spatial arrangement and leads to the synthesis of permissive ECM components^29^. Thus, 3D models enable the detailed analysis of the interaction between an active chemical substance and a biological system. This type of analysis is often required for *in vivo* toxicity analysis and drug screening. Recently, 3D cell-based anti-invasive and migration assays have gained tremendous interest in drug discovery because they can solve the known problem of discrepancies between the behaviour of cells on a flat substrate and *in vivo* ^30^. However, quantitative analysis and microscopic imaging of the morphodynamics during cell migration in a 3D tissue is technologically difficult. To address this challenge, we propose a toroid shaped tissue that is easily adaptable for 3D cell migration analysis using direct imaging of the inner opening closure.

At the molecular level, ligand and membrane receptors of cells in a 2D model are exposed evenly per unit area. In contrast, a 3D tissue has tightly packed clusters and more complex configurations^31^. Moreover, drug or soluble factors induce signal transduction and influence the cellular behaviour in three dimensions differently, because cellular topographic sensing is affected by spatial organization of the tissue^32^. Thus, in term of drug exposure, 3D models are obviously advantageous in predicting the response of cells to drug. To date, 3D cell migration assays are limited to embedding cells into a matrix^33–35^, fabrication of cells with biomaterials scaffolds^36–38^ or tissue ring closure using magnetic levitation^39^. The present paper reports a 3D model with toroidal geometry that is formed through self-assembly of cells.

Furthermore, we demonstrate here the ability of olfactory ensheathing cells (OECs) to migrate from a toroidal multicellular tissue by evaluating the rate of closure. OECs have been considered as the potential candidates for transplantation therapy following a spinal cord injury (SCI)^40,41^. The potential challenge of OEC-based therapy is enhancing the survival rate and the migration of OECs after the transplantation^42^. The migration of OECs is vital for the regeneration of neurons. As neurotrophic factors can promote the migration of OECs^43–45^, we employed glial derived neurotrophic factor (GDNF) in the experiments to promote cell migration and to evaluate the closure rate of the cell toroid.

## Concept of slow evaporation

**Figure 1:**
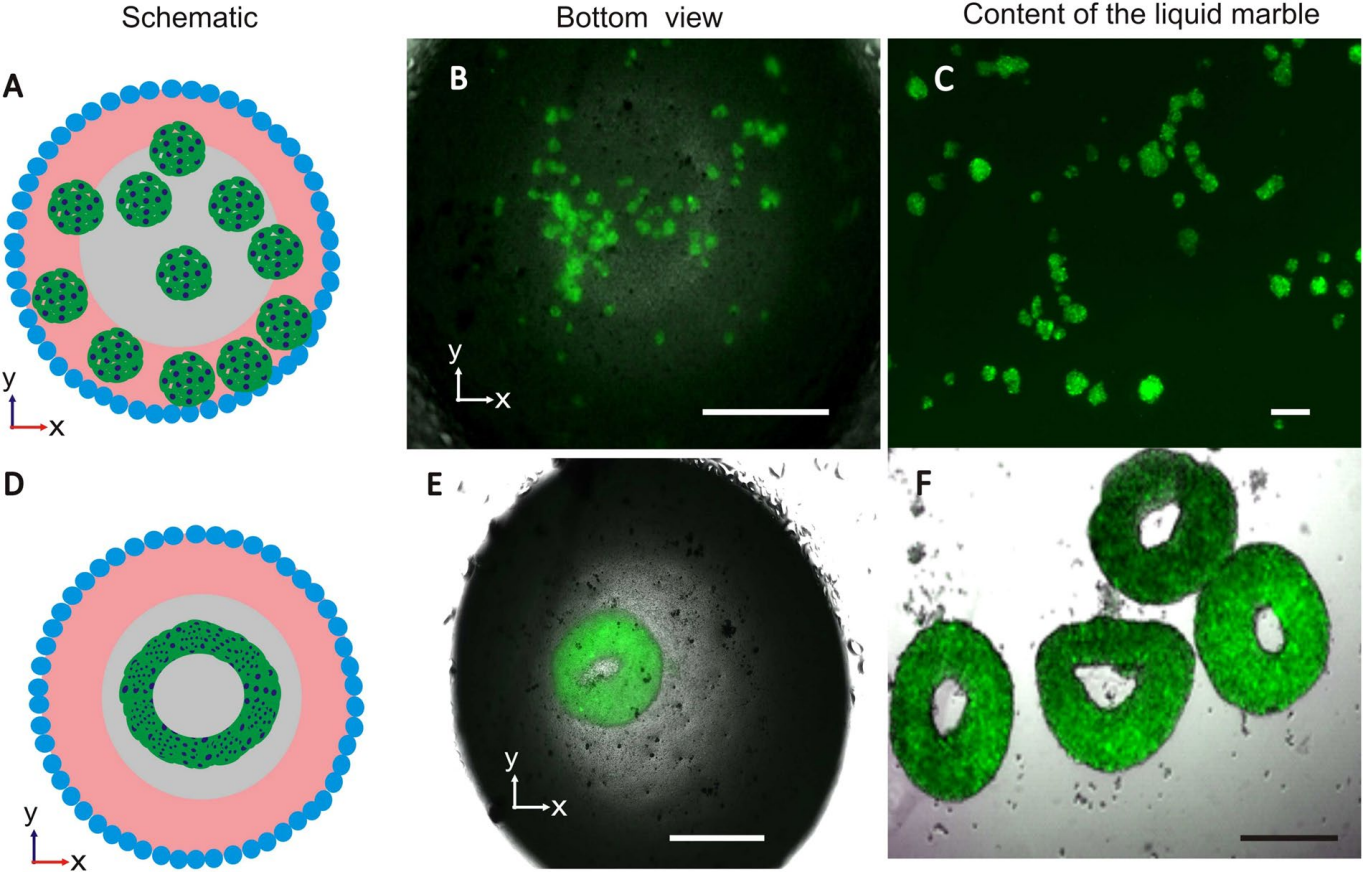
Physical properties of a liquid marble for the generation of spheroids and toroids (**A**) Schematic description of floating condition embedded with agarose hydrogel and the production of spheroids (bottom view). (**B**) Floating liquid marble producing multiple spheroids. (**C**) The morphology of spheroids harvested from several liquid marbles. (**D**) Schematic diagram showing toroid tissue generation inside a sessile LM (bottom view). (**E**) Toroid tissue formed inside a sessile marble. (**F**) The structure of toroids harvested from four sessile liquid marbles. Scale bar in (**A**) is 200 μm and (**B**,**E** and **F**) is 500 µm.

We address the evaporation problem by including a hydrogel sphere inside the sessile liquid marble. Preliminary experiments indicated that a floating liquid marble with a hydrogel sphere rapidly form spheroids, similar to the case of a floating liquid marble without embedded hydrogel^19^. A floating liquid marble behaves like a Leidenfrost drop^46^ and creates an internal flow that disperses the cells and let them interact freely to form multiple spheroids, Fig. 1A–C. In contrast, a sessile liquid marble on a solid surface generates toroid tissue. The formation of the toroid tissue is based on both chemotaxis and gravity driven self-assembly of cells around the bottom of the hydrogel sphere, Fig. 1D–F.

**Figure 2:**
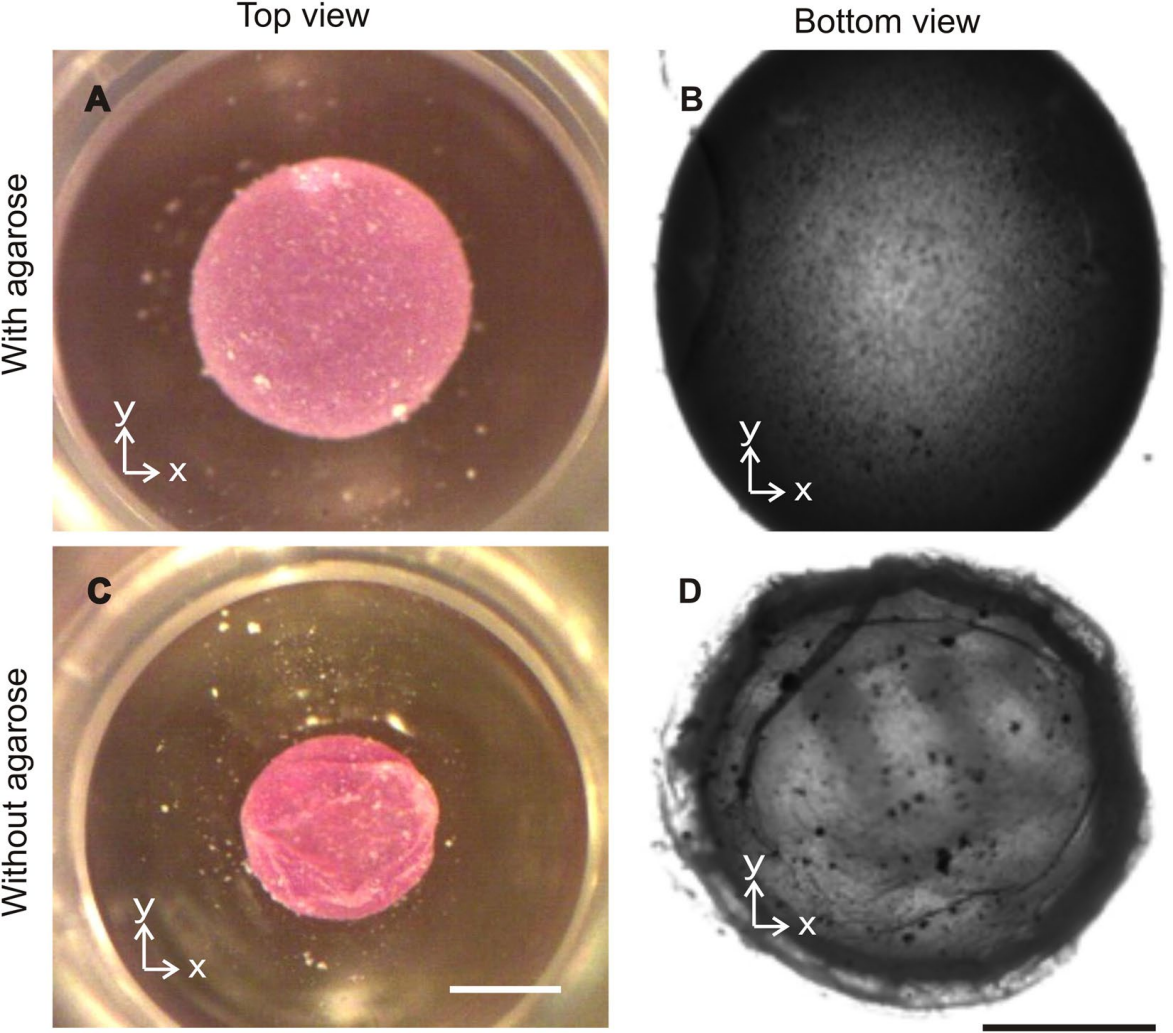
Effect of evaporation on a sessile liquid marble: (**A**) Top view of a sessile liquid marble with embedded agarose hydrogel. (**B**) Bright field image of a liquid marble containing agarose gel. The surface of the liquid marble did not show wrinkles. (**C**) Top view of a shrunken sessile liquid marble without agarose hydrogel. (**D**) Wrinkles on the surface indicating excessive evaporation. The total volume of the liquid in the marble is 10 µL. Scale bars are 500 µm.

Embedding a hydrogel sphere inside the liquid marble can slow down evaporation. A liquid marble containing a sphere made of agarose as the hydrogel maintains after 24 hours its spherical shape without significant wrinkles on the hydrophobic coating, Fig. 2A,B. In contrast, a sessile liquid marble lost its liquid content, shrunk and formed wrinkles after 24 hours, Fig. 2C,D. A sessile liquid marble suffers from excessive evaporation, which limits the life time of the cell culture. This phenomenon indicates that the embedded hydrogel sphere limited evaporation.

## Concept of slow release

We first numerically estimated the effect of diffusion in the liquid marble/hydrogel platform. The model predicts the concentration distribution of the growth factor inside the marble in three dimensions. The formation process of the toroid tissue is constrained in the space between the hydrogel sphere and the shell of the liquid marble. In the present work, we used epidermal growth factor (EGF), which is well characterized and known for promoting cell migration. Numerous previous studies have reported the use of EGF for *in vitro* release from encapsulated hydrogel^47–49^.

Taking the Fick’s diffusion law into account, the diffusion time of the growth factor in a spherical liquid marble, or the time to have the growth factor equally distributed inside the marble, can be estimated as^50^:

1 $${t}_{{\rm{d}}}={{r}_{{\rm{LM}}}}^{2}/6D$$

In our experiments, a 10-μL liquid marble has a radius of ${r}_{{\rm{LM}}}=1.34\,{\rm{mm}}$. Assuming a diffusion coefficient of the growth factor of *D* = 10^−7^ cm^2^/s^51^, the release time of the growth factor is estimated to be ${t}_{{\rm{d}}}\approx 8.27$ h. Thus, the cells should have enough time in the first few hours to aggregate around the high concentration next to the hydrogel sphere.

**Figure 3:**
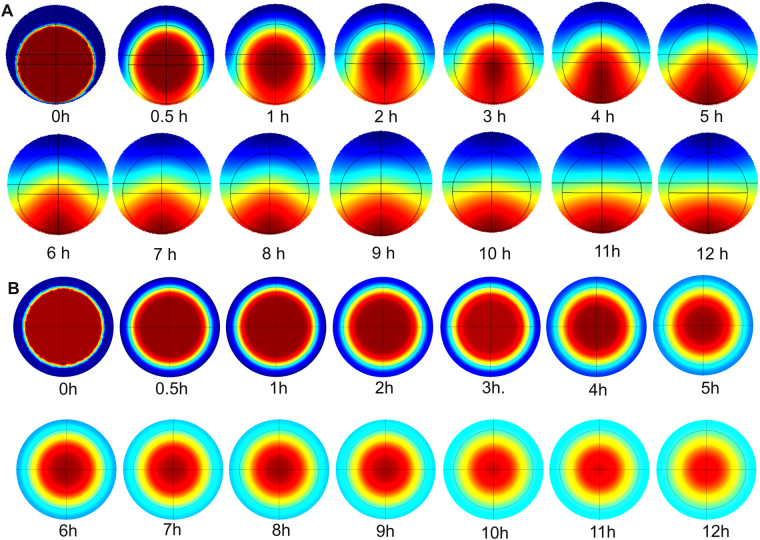
Simulated concentration distributions of growth factor over time inside the liquid marble through slow release from the embedded hydrogel sphere. (**A**) Volumes of the hydrogel sphere and the liquid marble are 3 μL and 5 μL, respectively. The diffusion coefficient of growth factor in both hydrogel and medium is assumed to be the same and has a value of *D* = 10^−7^ cm^2^/s. In the initial state (0 min), red indicates the hydrogel sphere; blue indicates the medium seeded with cells. (**B**) The same simulation was carried out for a hydrogel sphere positioned in the centre of the liquid marble. The radial concentration distribution is uniform.

Next, we conducted a 3D numerical simulation (COMSOL Multiphysics, COMSOL, Inc., USA) of the diffusion process of the growth factor to predict the location of the cell aggregates. Figure 3A shows the model and the corresponding concentration distribution over time. Red and blue colours indicate high and low concentration, respectively. The initial condition is the uniform concentration of growth factor inside the hydrogel sphere. The embedded hydrogel sphere is placed on the centre axis of a sessile liquid marble. The remaining space in the liquid marble is restricted by the hydrogel sphere and the non-adhesive shell (blue area in Fig. 3A, 0 min). This configuration represents the miniaturized 3D version of the conventional 2D “under agarose assay”. Figure 3A shows that the agarose sphere also serves as a reservoir to sustain the slow release of growth factor and water. The well-controlled high concentration of chemoattractant allows cells to migrate into a ring-like aggregate under the hydrogel sphere. According to the simulation, the higher concentration of growth factor at the bottom of the liquid marble can be maintained even after 12 hours of cell culture, when the tissue is ready to be harvested. As reference, the concentration field around a hydrogel sphere placed at the centre of the marble shows an expected uniform radial distribution over time, Fig. 3B. Thus, the distribution in Fig. 3A is unique for the sessile liquid marble used for growing the cell toroid described in the next section.

## Growing of toroidal tissue

**Figure 4:**
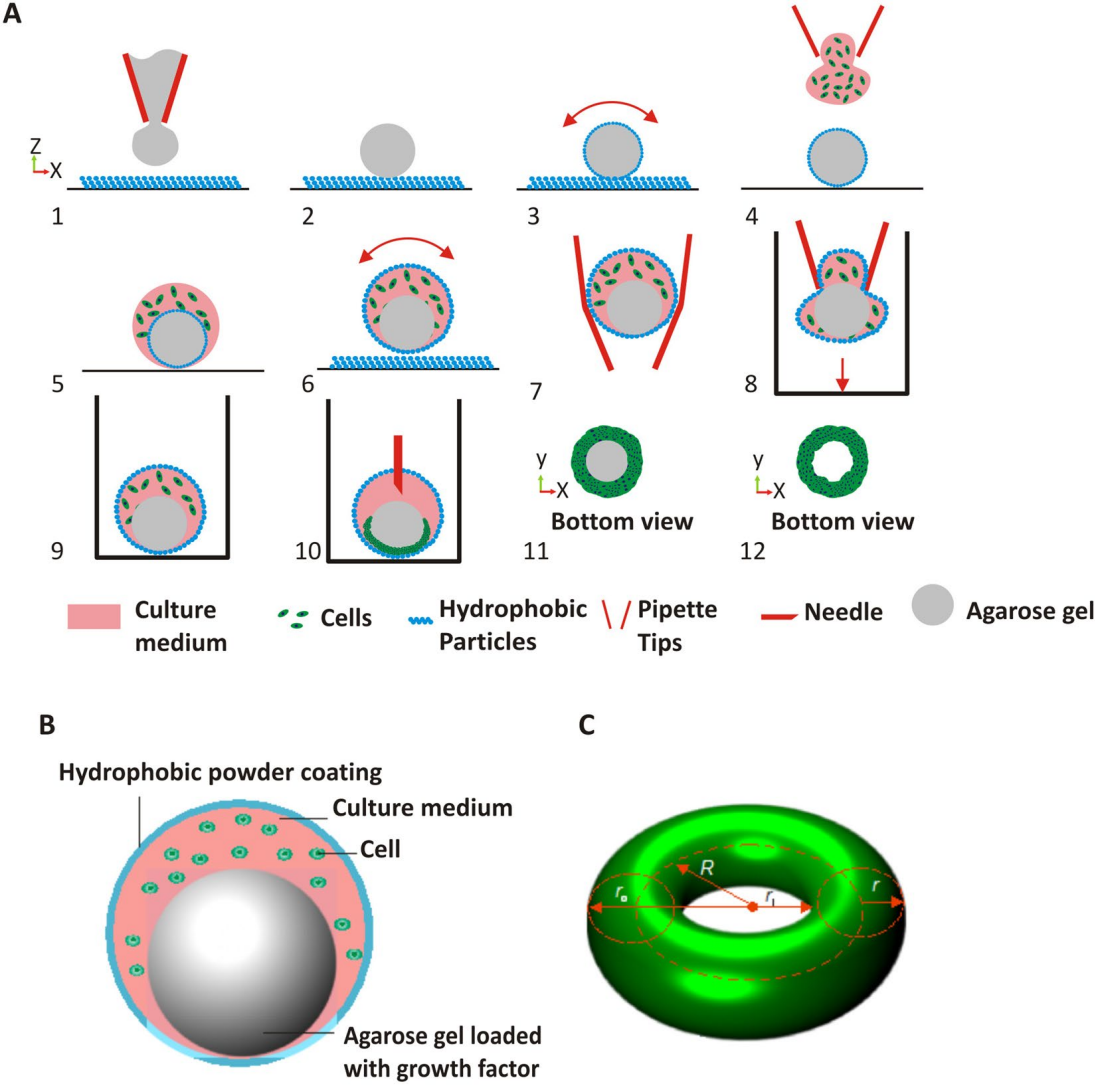
The bioreactor model, geometry of a toroid tissue and procedure for generating bioreactor platform for toroid tissue growth: (**A**) (1,2) Dispensing a hydrogel droplet on the powder bed. (3) Rolling the hydrogel droplet to coat it with hydrophobic powder. (4) Impacting an aqueous medium droplet containing cells on the hydrogel. (5) Encapsulating the hydrogel liquid marble with the medium and seeded cells. The hydrophobic powder of the hydrogel marble automatically migrates to the liquid/air interface of the larger droplet. (6) The composite liquid marble is rolled on the hydrophobic powder to ensure sufficient coating for maintaining its robustness. (7,8) The marble is picked up and transferred using a pipette tip and placed on 96 well plate. (9) A slow-evaporating liquid marble with cells and hydrogel for slow release of growth factor, ready for incubation. (10) After incubation, the cells settled and migrated at the bottom of the liquid marble to form toroidal tissue construct. (11) Marbles can be broken with needles to release the toroid tissue. (12) The toroid tissue and the agarose gel at the bottom of the well are separated for further analysis. (**B**) A liquid marble with embedded hydrogel sphere. (**C**) The basic geometry of a toroid (major radius *R*, minor radius *r*, inner radius *r*_i_ = *R* − *r*, volume $V=2{\pi }^{2}R{r}^{2}$, surface area $A=4{\pi }^{2}Rr$, aspect ratio $\alpha =R/r$) .

The key innovation of the platform reported here is the embedded hydrogel sphere. To start with, a 0.5% low melting (37 °C) agarose containing growth factors at 5-µL volume was coated with polytetrafluoroethylene (PTFE) powder with an average particle size of 1 μm (Sigma-Aldrich, product number 430935) by dispensing the gel onto the powder bed, Fig. 4A1,2. The agarose droplet was rolled in a circular motion to ensure that the PTFE powder covered the surface evenly, Fig. 4A3. After 3 to 5 minutes, the agarose droplet solidifies to form a sphere. This critical step ensures that the agarose spheres are consistent in size. Subsequently, a second droplet containing a predetermined number of OECs in the volume of 10 μL was injected to collide with the top of the agarose to form a single droplet containing cells and agarose, Fig. 4A4. By adjusting the pipetted volumes, liquid marbles with a volume ranging from 2 to 50 μL can be formed. For consistency, we kept the volume in this study constant at 10 μL. The hydrophobic particles on the agarose hydrogel is stable at the liquid/air interface. Once the contact between the liquid and the hydrogel is made (Fig. 4A5), the particles migrate toward the outer liquid/air interface. As a result, the hydrogel marble and the cell marble merge to create a composite liquid marble. Next, we gently rotated the composite droplet to create a robust liquid marble containing both cells and the agarose sphere, Fig. 4A6. Since the liquid marble is elastic, a cut 1000-μL pipette was used to handle the marble. The pipette was able to suck and hold the marble, Fig. 4A7. The marble was then dispensed into a U-bottom 96-well plate Fig. 4A8. The liquid marble was kept in the sessile condition for 12 hours and incubated at 37 °C, Fig. 4A9. The small particle size of the 1-μm PTFE powder creates a thin, porous coating, which allows for real-time observation of the assembly process of the cells. This protocol is suitable for the use of time-lapse microscopy to access the behaviour of toroidal tissue with different fluorescent labels. Finally, breaking the liquid marble (Fig. 4A10,11) releases the cell toroids which are subsequently seeded onto 96-well plates for further analysis Fig. 4A12. Figure 4B illustrates the schematic of the hydrogel/liquid marble bioreactor. Figure 4C shows the basic geometric parameters of the cell toroid, that were later used for its characterisation.

## Formation process of the cell toroid

**Figure 5:**
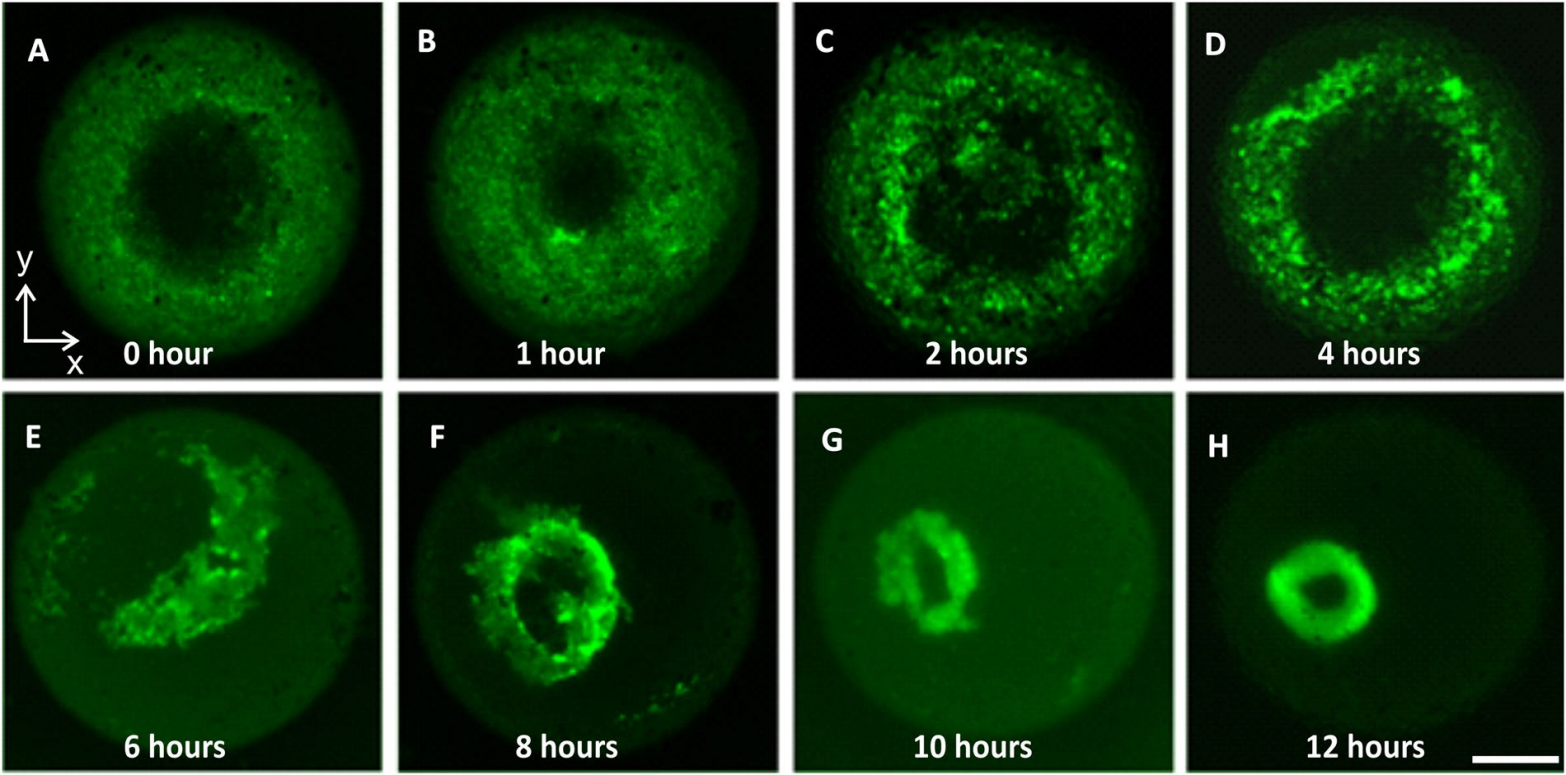
The formation of toroidal tissue inside liquid marble over time. Scale bar is 500 µm.

Next, we investigated the mechanism that governs the self-assembly of cells to form the cell toroid, particularly to understand the relationship between tissue shape and tissue deformation. We observed the cell-cell interaction by imaging the liquid marble at different instances. After about 30 minutes, the cells aggregate at the bottom of the liquid marble due to gravity and chemotaxis, Fig. 5A,B. The cells assembled at the lower surface of the liquid marble. Subsequently, between 2 and 4 hours, the cells attract each other and aggregate into a ring shape around the agarose sphere, Fig. 5C,D. Within 6 to 8 hours, the cell aggregates underwent two steps. In the first step, cell aggregates migrate underneath the agarose towards the bottom of the liquid marble, Fig. 5E. During this time, the inner shape of the toroid was still not completed, Fig. 5F. The second step is the self-assembly of cells into small unit blocks, which subsequently fuse into a thin toroid, Fig. 5G. Between 10 and 12 hours the tissue contracted significantly and formed a perfect toroid, Fig. 5H. Cell assembly and compaction under the surface tension contributes to the formation of the toroidal shape, which has a perfect circular opening.

## The role of seeding cell concentration

**Figure 6:**
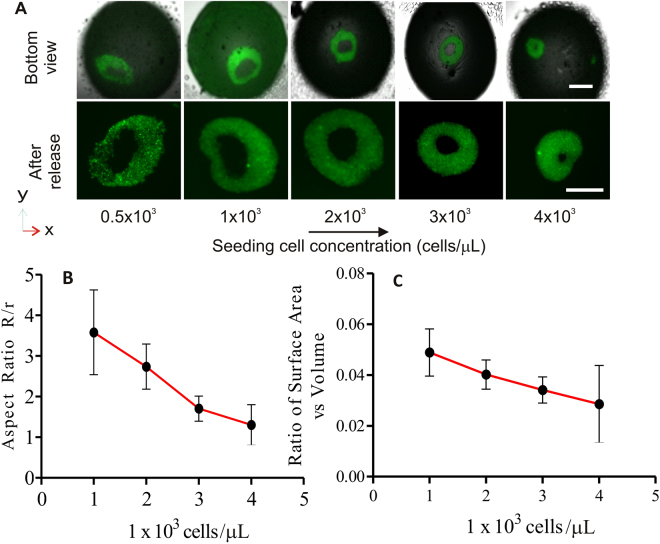
The role of seeding cell concentration: (**A**) Toroids with different seeding cell concentrations. (**B**) Aspect ratio as function of seeding cell concentration. (**C**) Surface area to volume ratio as function of seeding cell concentration. All experiments were repeated three times with n = 5 toroids each. Bars represent the mean; error bars represent the standard error of the mean.The scale bar inside the liquid marble is 500 µm and the harvested toroid is 200 µm.

We next quantified the effect of seeding concentration on the formation of a cell toroid. The ability of cells to self-assemble into a toroid depends on the number of viable cells and the concentration of the growth factor. We performed experiments with a fixed volume of 10 µL medium and 5 µl of 0.5% agarose solution, to determine the optimal cell concentration for the geometry of the final toroid. Cells were seeded with concentrations ranging from 0.5 × 10^3^ to 4 × 10^3^ cells/µL. The tissue was allowed to assemble for 12 hours, Fig. 6A. The liquid marble was subsequently broken to release the cell toroids for measuring the geometric parameters such as the inner radius *r*_i_, the minor radius *r*, and the major radius *R*, Fig. 4C.

A concentration of 1 × 10^3^ cells/µL resulted in a thin toroid and a large surface area to volume ratio. Eventually, cell concentration of less than 1 × 10^3^ cells/µL did not result in an uniform distribution. Therefore, the cells aggregated into irregular shapes or thin toroids that are prone to rupture. Figure 6B shows the aspect ratio *R*/*r* as a function of the cell concentrations, indicating a thicker toroid with a larger minor radius *r* and an almost constant major radius *R*. As the major radius *R* is determined by the constraining geometry of the liquid marble and the agarose sphere, this parameter does not vary with the concentration of the cells. However, the minor radius *r* depends on how fast the cells assemble and grow, thus dependinh on the cell concentration. A higher cell concentration results in a higher growth rate and a thicker toroid over the same culturing period. This result indicates that a significant number of cells are crucial for adjacent cells to self-assemble into a toroid.

Figure 6C depicts the relationship between the ratio of surface area versus volume and the cell concentration. The surface area and volume are calculated according to the geometry of a torus depicted in Fig. 4C. The outer radius of the toroid did not significantly vary with increasing cell concentration, suggesting that the space between the hydrogel sphere and the shell determines the outer shape of the toroid. The inner radius *r*_i_ decreases with increasing cell concentration leading to a lower surface area to volume ratio. At a concentration of 5 × 10^3^ cells/µL, the cells migrate, compact and take up a spherical shape. The seeding concentration of 2 × 10^3^ cells/µL produces cell toroid with a surface area to volume ratio of approximately 0.04.

**Figure 7:**
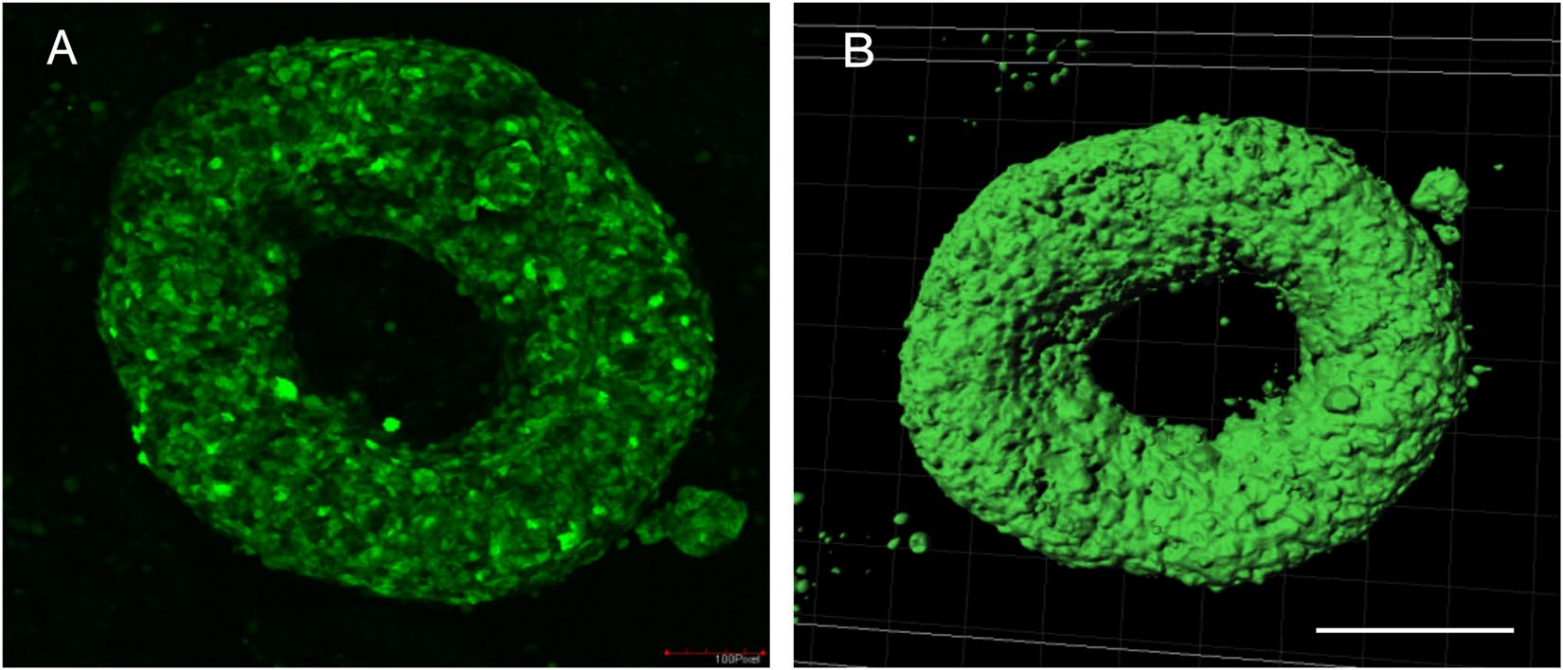
Morphology of a cell toroid: (**A**) A single confocal slice of the toroid. (**B**) The assembled 3D image indicates a toroidal shape. Scale bar is 200 µm.

The criteria for an ideal toroid are as follow. (i) The toroid should have the largest surface area per unit volume for making efficient diffusion of nutrient and effectively dispel waste from the inner core. (ii) The opening should be symmetric because its diameter is critical for the use of the toroid as a 3D wound healing model. At a lower cell density (0.5-1 × 10^3^ cells /µL), we obtained toroid tissue with a higher surface area per unit volume but the tissues were irregular in shape. Further increasing the cell density to 3 and 4 × 10^3^ cells /µL produces thicker toroids with a smaller inner radius. A thicker toroid may affect the tissue viability. Thus, in our subsequent experiments, the optimal number of cells necessary to display a symmetrical, stable and functional toroid is 2 × 10^3^ cells /µL, Fig. 7A,B.

## The role of agarose concentration

**Figure 8:**
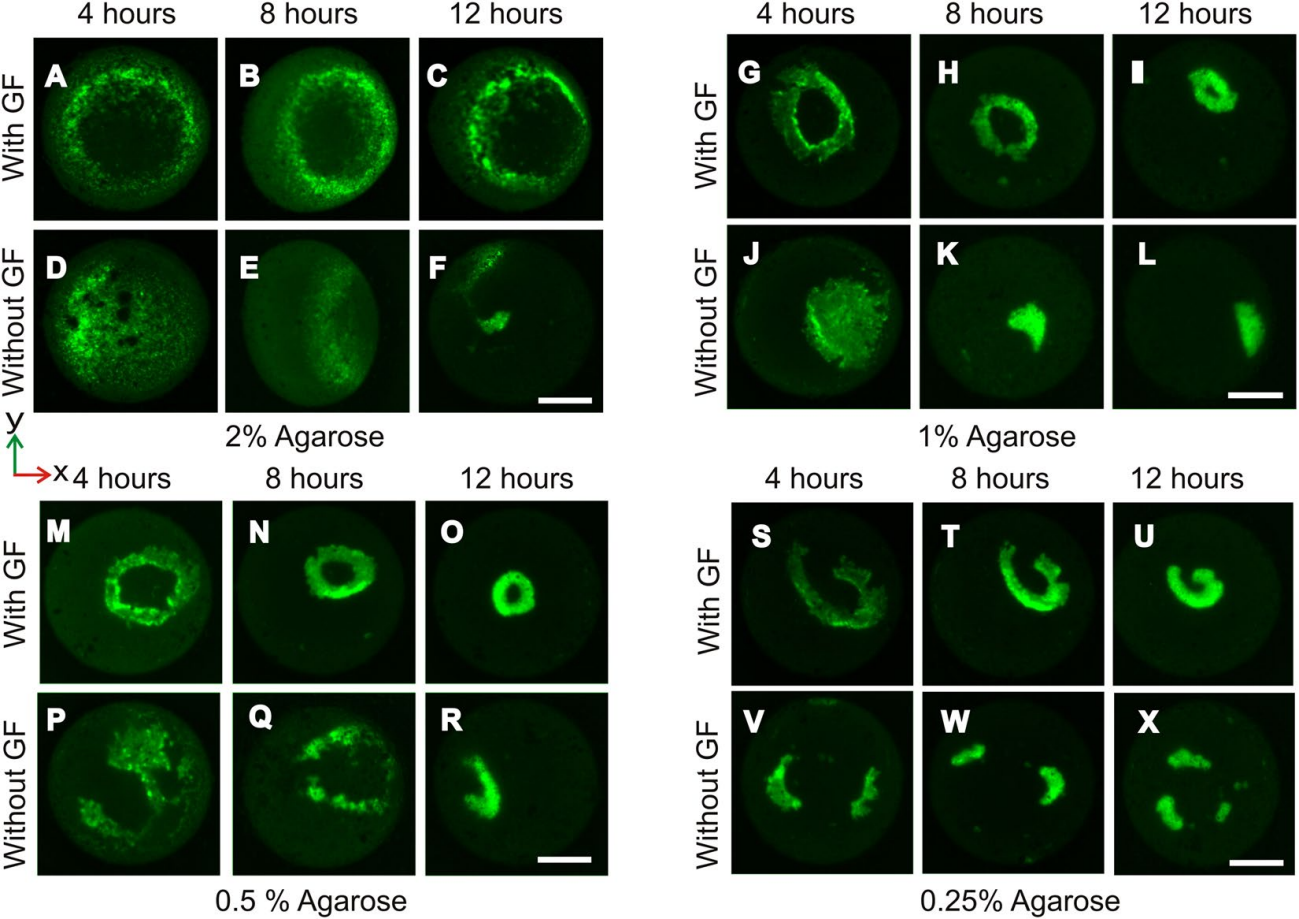
The role of agarose and growth factor in toroid formation. The release of growth factor and cell assembly to form of cell toroids from 4 to 12 hours (**A**–**C**). Toroid formation was disrupted at 2% agarose with growth factor. Irregular toroidal tissues are formed at 1% agarose (**G**–**I**) and at 0.25% agarose (**S**–**U**). The ideal toroid was formed with 0.5% agarose containing growth factor (**M**–**O**). Cells did not assemble into toroid in all conditions without growth factor. The scale bar is 500 µm.

We created agarose spheres with a concentration between 0.25 and 2% with and without growth factor, to elucidate the contribution of agarose in the formation of the toroidal tissue. Particularly, we aimed to determine whether the toroid formation is caused by cell sedimentation at the bottom of the liquid marble. The formation of the toroid was observed with these agarose spheres at 4, 8 and 12 hours, Fig. 8. Surprisingly, cells did not assemble to form toroid in the absence of growth factor under all conditions. Furthermore, a high concentration of agarose of 2%, eventually failed to create a cell toroid even with growth factor, Fig. 8A–C. The cells did not migrate under the agarose and exhibit a poor rate of cell assembly, especially at 12 hours. Cell aggregates were not uniformly distributed in a circular pattern at 4 hours in the absence of growth factor.

Interestingly, cells assembled under the agarose sphere to form the toroidal shape at agarose concentrations of 0.5 and 1%. However, we did not observe any obvious differences between these concentrations. An ideal toroidal shape was observed with the agarose concentration of 0.5%, Fig. 8M–O. A lower agarose concentration of 0.25% results in an irregular toroidal shape with a larger inner radius, Fig. 8S–U. This observation further supports the role of agarose in stimulating cell aggregation and assembly by the slow release of growth factor through the diffusion process. The release process depends on the concentration of the agarose and the corresponding porosity. The pore size of an agarose gel increases with decreasing concentration. Thus, 0.5% agarose is optimal for diffusion. Higher or lower agarose concentrations may release growth factor too slow or too fast for the required cell assembly.

The sedimentation of cells inside the LM and the subsequent formation of cell toroids are facilitated by the following mechanisms: (i) gravity enforced assembly of cells, (ii) cell-cell cohesion, (iii) chemotaxis due to growth factor release from the agarose. After an initial incubation time, the seeded cells settled at the bottom of the liquid marble. Subsequently, the cells aggregate and assemble into a ring shape around the lower region of the agarose sphere. In the absence of growth factor, cells aggregate into irregular clusters. As shown in the numerical simulation, the growth factor concentration is higher at the lower hemisphere of the liquid marble. The high concentration of the growth factor direct the cell migration and enhance cell-to-cell adhesion around the bottom of the agarose gel. As a result, cell–cell interactions contribures to cell contraction and self-assemble into a toroid. We also found that the opening of the toroid did not match that of the diameter of the agarose sphere. In fact, the diameter of the opening decreases with increasing cell concentration. Thus, we hypothesise that the contribution of the growth factor to the formation of the toroid dominate over the gravity effect and the surface properties of the agarose.

## Closure of the 3D toroidal tissue

**Figure 9:**
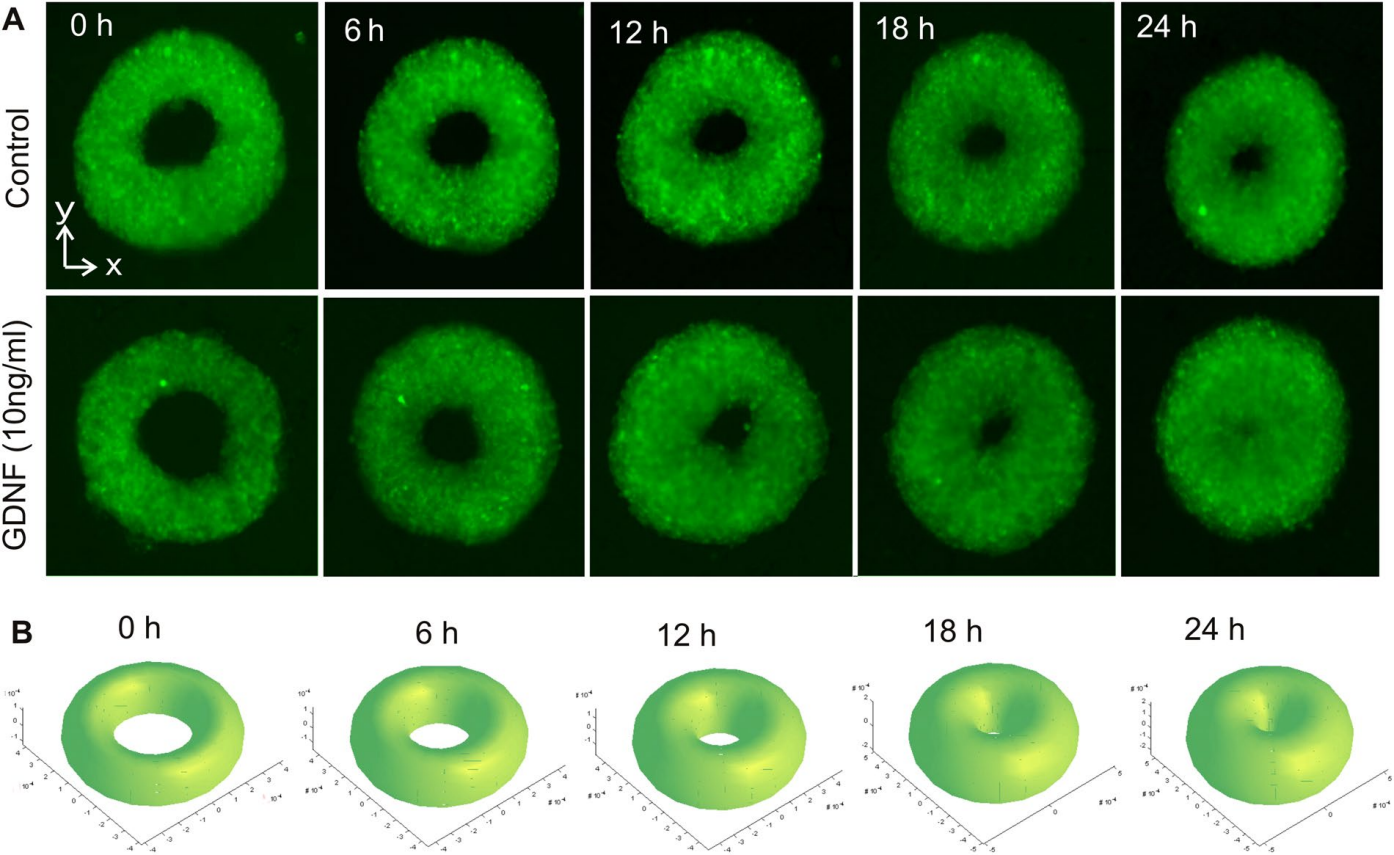
The effect of glial cell-derived neurotrophic factor on the closure process of a cell toroid: (**A**) Toroidal shapes over time. (**B**) The geometric model of the closure process with a constant growth rate (*R* = 300 μm, *r*_0_ = 120 μm, *k* = 5 μm/hr). Scale bar is 200 µm.

We use a geometric growth model to predict the tissue closure based on a constant linear growth rate. The measure of lumen closure is the instantaneous value of appreciable cell migration, which models the mechanism of wound closure in three dimensions. Figure 9 shows the closing process of a cell toroid after harvesting them from the liquid marble/agarose bioreactor. Figure 9A indicates that glial cell-derived neurotrophic factor (GDNF) increases the percentage of inner circumference closure at a time instance between 6 to 24 hours as compared to the control. The data indicate that the closure of toroid lumen progressively increase from 0 to 12 hours, suggesting that in early stage cells actively migrate instead of proliferation. Subsequently, the closure rate decreased, and complete lumen closure occurred between 18 to 24. At this stage, the cell population may involve both cell migration and proliferation. Previous investigation indicated that mitotically active cells depends on cell doubling time, which are prominent and persist 24 h after wounding^52^. Moreover, the outer diameter of the toroid in our study decreases over time and did not show evidence of outward spreading. These observations allow us to hypothesise that cell migration is actively involved, evidiently through the persistent closing of the lumen. In addition, the wound closure rate is chemotactically driven by GDNF, which is known to induce OECs migration^44^, but not proliferation^53^. In this regards, cell migration can be the dominant mechanism, and cell proliferation is assumed to be a secondary event during the lumen closure process.

The geometric model for interpreting the experimental data of Fig. 9A assumes a linear growth rate in the radial direction of the minor radius *r(t)*:

2 $$r(t)={r}_{0}+kt$$

where *r*_0_ is the initial minor radius of the toroidal tissue and *t* is the time. The growth rate *k* depends on the culture medium, e.g. the concentration of the growth factor and the cell concentration. The existence of growth factor contributes to a higher growth rate. The time dependent shape of the surface of the toroid can then be described based on the geometry as follows:

3 $$x(t)=[R+r(t)\cos \,\theta ]\cos \,\phi $$

4 $$y(t)=[R+r(t)\cos \,\theta ]\sin \,\phi $$

5 $$z(t)=r(t)\sin \,\theta $$

where $\theta$ and $\phi$ are the rotating angle around the minor and major axis of the toroid, respectively. Figure 9B shows the closure process of a representative toroidal tissue with a major radius *R* = 300 μm, initial minor radius *r*_0_ = 120 μm and a grow rate of *k* = 5 μm/hr. The results indicate that the geometric model with a linear growth rate describes well the observed process depicted in Fig. 9A. To confirm the linear model, we evaluated the geometric parameters of the toroidal tissue for both control and GDNF over time.

**Figure 10:**
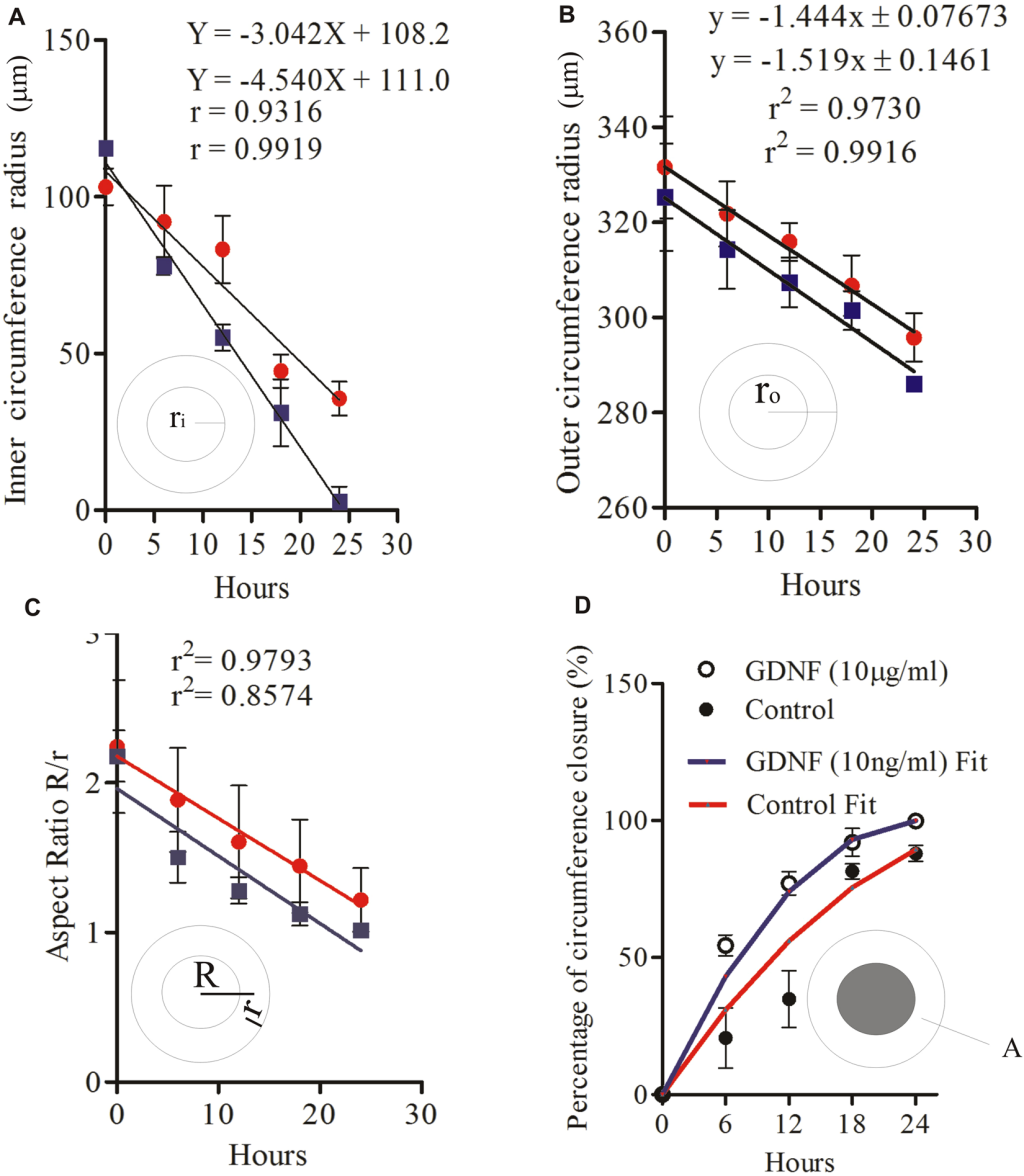
The effect of GDNF on the geometric parameters of the toroid. (**A**) Inner radius over time. (**B**) Outer radius over time. (**C**) Aspect ratio over time. (**D**) Percentage of cavity closure areas over time. Lines are fitting functions based on the respective linear models of (4), (7) and (8). All experiments were repeated three times with n = 3 toroids each. Bars represent the mean; error bars represent the standard error of the mean.

Figure 10A shows the inner radius ${r}_{i}=R-r\,\,$or the cavity radius as a function of time. A clear linear behaviour could be observed in both control and GDNF. Figure 10B shows the outer radius of the toroid as a function of time, indicating the overall shrinking of the cell toroid. In contrast to the linear growth model, the outer radius of the toroid decreases linearly over time, indicating continuous compaction of the tissue. Linear behaviour is reflected well in the aspect ratio of the toroidal tissue, Fig. 10C. Assuming that a cell toroid with an inner radius of ${r}_{i,0}={(R-r)}_{0}\,\,$grows in a radial direction at a constant rate *k* (Equation 3), the function of the inner radius over time *t* is:

6 $${r}_{i}(t)={r}_{i,0}-kt$$

Thus the cavity closure as a function of time can be estimated as:

7 $$\alpha =\frac{{A}_{0}-A(t)}{{A}_{0}}\times 100 \% =[1-{(1-kt/{r}_{i,0})}^{2}]\times 100 \% $$

where ${A}_{0}\,\,$ and $A(t)\,\,$ are the initial cavity area and the time dependent cavity area, respectively. The inner radius grows radially inward at a constant rate *k* (Equation 3). The closure rate *k* can be determined from the measurement of the inner radius and a linear fit over time. The closure rates of the control experiments and experiments with GDNF are 3.04 μm/hr and 4.54 μm/hr, respectively. As mentioned above, the outer diameter of the toroid decreases over time, but at a lower rate than the closure rate. The outer radius decreases at a rate of 1.44 μm/hr and 1.52 μm/hr for control and GDNF, respectively, Fig. 10A,B. The reason for the shrinking behaviour of the outer diameter could be the compaction of the tissue to minimize its overall surface area as a result of the closure. Figure 10D shows the evaluated cavity closure (7) of both cases over time. Clearly, GDNF has contributed to a faster closure of the cell toroid.

## Discussion

First, we demonstrated the use of a liquid marble with embedded hydrogel as an ideal bioreactor platform to engineer cell toroid and its application as a 3D wound closure assay. The embedded hydrogel maintains the water content critical for culturing cells. Most microfluidic cell culture platforms use a small volume of liquid, and therefore evaporation is a major problem even in a humidified environment. Moreover, evaporation in a small volume affects the osmolality of the extracellular environment that alters the biochemical balance and impedes cell growth^54^. The hydrophobic and porous shell of a liquid marble is permeable, allowing for the gas exchange that is vital for cell culture. At the same time, this porous shell makes it prone to evaporation. Our system uses a hydrogel sphere as the water storage^55^ to reduce evaporation.

Second, the hydrogel sphere facilitates growth factor release and attracts cells to settle on the bottom of the liquid marble in a ring. In an aqueous environment, hydrogel swells by absorbing water and simultaneously release loaded solutes. Their porosity permits loading of the drug or growth factor. The released molecules diffuse to the surroundings, allowing slow drug release^56,57^. Our optimization experiments indicated that a liquid marble seeded with 2 × 10^3^ cells and 0.5% agarose containing growth factor resulted in an ideal toroid shape in just 12 hours. Hydrogels with larger pores cause premature dissolution or rapid drug release in a shorter time span. Our experimental data confirm this hypothesis. At an agarose concentration of 2.5%, we did not observe toroid tissue formation. Furthermore, at this concentration, the agarose may deform and do not maintain a spherical shape due to the lower interfacial tension relative to its weight.

Third, the bioreactor platform is sufficiently sensitive for the assessment of chemotaxis induced self-assembly of cells. Chemotactic stimuli display signal transduction events that activate directed cell migration as collective units^58,59^. An organized migration toward external cues is critical for cells to aggregate into a uniform ring-like pattern, only 4-6 hours after seeding. In a liquid marble containing agarose without growth factor, cells are not able to migrate effectively. Experimental data show a decrease in cellular cohesion and non-homotypic aggregation. This observation evidently validates the hypothesis that cells undergo cluster attraction and migrate along the gradient of the growth factor. We formulated and carried out a numerical simulation of the concentration distribution of growth factor over time and found an agreement with chemotaxis-driven cell aggregation in our experiments. In addition to chemotaxis, cytoskeletal mediated tension/contraction contributes directed self-assembly to form a complex toroidal microtissues^60^.

Fourth, our bioreactor platform allows for the regulation of the size of a cell toroid using the seeding concentration. Appropriate seeding concentration is crucial for obtaining the ideal toroid geometry due to the higher assembly or growth rate. Non-axisymmetric toroidal shapes are possible at low cell concentration. In contrast, a lower aspect ratio of approximately 1.0 was observed at a higher seeding concentration. The observation confirms that seeding concentration influences the migration and aggregation dynamics. Geometric parameters of cell toroid such as volume, surface area, minor and major radii are critical for tissue viability and functionality. For instance, a toroid with a large surface area to volume ratio is desirable, as sufficient nutrient diffusion and supporting metabolites can reach the interior milieu of intact cells. Moreover, enhanced solubility and stimuli of growth factor are mediated by abundant cell-surface receptors, which promote cell signalling and tissue growth. Interestingly, we identified a toroid with surface-area-to-volume ratios as high as 0.04 at a cell concentration of 2 × 10^3^ cells/µL could conserve appropriate function for wound healing or 3D cell migration assay.

Finally, we demonstrated that the cultured cell toroid serves well as a 3D wound closure assay. Rapid closure of the toroid inner circumference was achieved if the tissue was treated with 10 ng/ml growth factor. Experimental data agreed well with the model of a constant closure rate. Toroidal tissues treated with growth factor clearly showed a higher closure rate than that without growth factor.

### Outlook

The bioreactor platform reported here is useful for studying drug stimuli on cell sorting, cell-cell interaction and self-assembly to engineer the 3D unit of a more complex tissue structure. In addition, this technology could potentially be used to test different biomaterials in conjunction with drug eluting strategy to engineer tissue formation. Further work is needed to scale up this technology to large-scale production of cell toroids suitable for various applications ranging from drug discovery to 3D bio printing.

## Materials and Methods

### Cell culture

The green fluorescent protein (GFP) expressing immortalized mouse OECs were obtained from Prof Filip Lim, Universidad Autonoma de Madrid, Madrid, Spain. Cells were cultured in DMEM/F12 (Life Technologies) supplemented with 10% FBS (vol/vol), 2 μM forskolin (Sigma), 20 μg ml^−1^ pituitary extract (Gibco), 10ng ml^−1^ FGF-2 (PeproTech), 10ng ml^−1^ EGF (PeproTech) and 0.5% (vol/vol) gentamicin (Life Technologies). Cells at 75% confluence were harvested from in T25 flasks by washing twice with HBSS (Life Technologies) and detached with TrypLE Express (Life Technologies) for 5 minutes at 37 °C. Subsequently, the enzymatic reaction was stopped using 2 mL 10% FBS media and the solution centrifuged at 1000 rpm for 5 min. The OECs were grown by changing media every 2 days.

### Preparation low–melting point hydrogel

In the experiments of this paper, agarose serves as the hydrogel. An amount of 0.01 g low-melting-point agarose (Invitrogen) was placed into a 10-mL falcon tube and diluted into 10 ml HBSS to make a 1% agarose solution by careful heating with microwave oven until boiling, stirred to facilitate complete dissolution. When the temperature decreased to 40 °C, the agarose solution was further diluted in 1:1 ratio with a medium, or medium containing 10 ng/mL epidermal growth factor (EGF), and suspended in the 1.5-mL Eppendorf tube. The Eppendorf tube was heated at 37.5 °C using a hot plate inside a hood. The mixture was heated on a hot plate in the cell culture hood until boiling. Subsequent stirring facilitates the complete dissolution of the agarose. Most experiments in this paper utilized 0.5% agarose.

### Preparation of hydrogel sphere

Polytetrafluoroethylene (PTFE) powder with particle sizes of 1 μm (Sigma-Aldrich, product number 430935) was prepared inside a 6-well plate. A micropipette was then used to dispense 5 µL of 0.5% agarose on the hydrophobic powder bed. The droplet was gently shaken in a circular motion for 2 minutes to form a hydrogel liquid marble. The hydrogel liquid marble was then kept for 3 minutes to ensure agarose completely solidifies to form the hydrogel sphere.

### Preparation of encapsulated hydrogel sphere with cells

A pre-determined number of cells (1000 to 5000 cells/μL) in 10 µL were dispensed vertically on top of a sessile hydrogel liquid marble on a PTFE powder bed. The PTFE particles coated on hydrogel disperse and migrate out to the liquid-air interface, once merging with the second droplet containing cells. Finally, the compound droplet was rolled on the powder bed in a circular motion for two minutes to form the lager liquid marble containing both cells and the hydrogel sphere. Figure 1A shows the bioreactor system with a hydrogel sphere embedded in the liquid marble.

### Toroid geometry measurement

We calculated the aspect ratio and the volume using the values of major (R) and minor (r) radii of the toroid. The radii values were determined based on average value obtain from ten measurements from each toroid tissue. The total number of toroids used in each group were n = 5. We used the equation $V=2{{\rm{\pi }}}^{2}R{r}^{2}$ and ${\rm{A}}=4{{\rm{\pi }}}^{2}Rr$ for for calculating the volume and area, respectively. The aspect ratio is defined as *R*/*r*. All measurements were carried out using ImageJ software.

### Cell toroid closure assay

For three-dimensional wound closure assays, individual cell toroid was harvested from the liquid marble. Subsequently, a media volume of 50 μL was dispensed using p1000 pipette tips into each well of a 96-well plate containing broken liquid marble followed by careful suspension and picking the cell toroids. Harvested cell toroids were transferred into a 384-well plate with non-adherence surface. Finally, a media volume of 50 μL containing 10 ng/mL GDNF or test drugs were dispensed into the well. Bright field and fluorescent images were obtained using a microscope (Olympus IX70) equipped with the Axiocan camera. Fluorescent images were captured at 6-hour intervals for 24 hours. Images were analysed using ImageJ software (NIH, USA).
